# Exploring emergency department visits: factors influencing individuals’ decisions, knowledge of triage systems and waiting times, and experiences during visits to a tertiary hospital in Saudi Arabia

**DOI:** 10.1186/s12245-019-0254-7

**Published:** 2019-11-21

**Authors:** Nawaf Alhabdan, Faisal Alhusain, Abdulkareem Alharbi, Muatassem Alsadhan, Moath Hakami, Emad Masuadi

**Affiliations:** 1 College of Medicine, King Saud bin Abdulaziz University for Health Sciences, Riyadh, Saudi Arabia; 2 King Abdullah International Medical Research Center, Riyadh, Saudi Arabia; 3 Department of Emergency Medicine, Ministry of the National Guard - Health Affairs, Riyadh, Saudi Arabia

**Keywords:** Triage knowledge, Emergency medicine, Primary health care, Patient preference

## Abstract

**Background:**

In recent years, there has been an increased utilization of emergency departments (EDs) in many countries. Additionally, it is reported that there are major delays in delivering care to ED patients. Longer waiting times are associated with poor patient satisfaction, whereas an understanding of the triage process increases satisfaction. This study aimed to assess ED visitor’s awareness of the triage procedure and their preferences regarding delayed communication.

**Methods:**

Cross-sectional study of King Abdulaziz Medical City – Emergency Department visitors using a previously validated questionnaire (Seibert 2014) which was translated to Arabic, piloted, and then used for this study.

**Results:**

A total of 334 questionnaires were returned. The mean age of respondents was 33 years. Regarding primary care physicians, only 16% of respondents said that they have one. About 21% of those tried to communicate with them before coming to the ED. Even though only 11% of respondents knew exactly what triage is, 51% were able to correctly explain why some patients are seen before others. Statistical analysis did not show any factors that are associated with increased knowledge of triage. Most respondents (75%) want to hear updates regarding delays with 69% of them preferring to be updated every 30 min.

**Conclusions:**

This study showed that the majority of patients do not know what triage means and that most of them want to know how the ED works. Moreover, a lot of respondents said that they do not have a primary care physician. These results support increasing patient awareness by education and involving them if any delay happens.

## Introduction

In recent years, there has been an increase in the utilization of emergency departments (EDs) in many countries, including Saudi Arabia, as the number of patients keeps rising [1, 2]. Such an issue can be partly attributed to non-urgent presentations, which comprised approximately 50% of all ED cases in Saudi Arabia and Australia [3, 4]. In Saudi Arabia, non-urgent patients reported multiple reasons for visiting the ED including the lack of a regular primary healthcare provider, convenience and quick access, and the perception that they will receive better care [5]. In addition, major delays in delivering care to patients in EDs have been reported in Riyadh (the capital city of Saudi Arabia) [6]. All these factors can lead to ED overcrowding with consequences such as increased waiting time, impaired patient-centered care, and disrupted evaluation and treatment [7].

Another component of this issue is triage, which is the assignment of priority to patients based on the level of urgency attached to their case [7]. The main goal of triage is not to increase or decrease general waiting times, but to provide immediate care for those in most need [8]. Therefore, triage leads to decreased waiting time for all patients except those in the lowest priority category [9]. Long waiting times are not only associated with poor satisfaction, but can also negatively impact patients’ perception of information, instructions, and the overall treatment [10, 11]. Decreasing waiting times is not an easily achievable aim; however, factors such as improving patient knowledge of how the assignment procedure works and communication of estimated waiting times can increase patients’ satisfaction during ED visits [10, 12, 13].

Additionally, patients’ expectations regarding waiting times for laboratory or imaging results (conducted in the ED) appear to vary greatly. In one study, the interquartile range (IQR) for patients’ time expectation for computed tomography (CT) scan results, for example, was 30 to 94 min [14]. This variation could be attributed to a general lack of knowledge regarding such procedures.

A review of the literature revealed some studies that addressed patient awareness of ED triage. For example, Adeniji et al. reported that patients lack a good understanding of the triage [15]. Meek et al. reached a similar conclusion and added that patients wanted to know more about the process [16]. Additionally, Seibert (2014) found that only 33% of patients were aware of the definition of triage [14].

To our knowledge, no studies have yet been conducted to evaluate ED patients’ knowledge of triage in Saudi Arabia or the Middle East. Therefore, in this study, we aimed to assess ED visitors’ knowledge of triage, to understand the factors influencing individuals’ decisions to visit an ED and to explore the experiences of ED visitors to a tertiary hospital in Riyadh, Saudi Arabia.

## Methods

### Study setting, subjects, and design

This cross-sectional study was conducted at the adult ED at King Abdulaziz Medical City, Riyadh, Saudi Arabia, which is a tertiary care hospital with a bed capacity of 1501 and a level I trauma center that receives approximately 220,000 ED visits each year. The ED follows the Canadian triage system which has 5 levels. Patients in the waiting area and meeting the inclusion criteria (classified as level 3, 4, or 5, age > 14 years, Arabic speakers, and cognitively competent) were enrolled; patients who did not meet the inclusion criteria were excluded. In total, 334 patients participated in this study by filling a printed data collection form (DCF) during their waiting time.

### Data collection form

A 31-question DCF was adopted from a previous published study conducted in the USA [14]. To meet the requirements of our study subjects, the questionnaire was translated into Arabic. The survey was composed of two major sections. The first was designed to obtain demographics information such as age, sex, education level, and marital status. The second section focused on the use of primary health care facilities prior to the patient’s ED visit, their understanding of ED triage system, the desire to be informed about the delays as well as common health problems (e.g., diabetes and heart attacks) during the waiting time, and the time patients expect to wait for results from some ED services, such as X-rays or CT scans.

### Sample size

Based on a literature review which estimated that 33% of the population were aware of the triage system, the margin of error and the confidence level of this study were 5% and 95%, respectively, and a minimum sample size of 334 was required to achieve statistical power [14].

### DCF piloting

In general, DCFs were filled within 5–15 min. This was dependent on the participant’s answers (some “yes” answers required responses to additional questions), participant’s enthusiasm (some intentionally answered “no” to avoid answering additional questions, or skipped some questions), and sometimes being called for treatment while filling the questionnaire. The team tried to overcome these obstacles by ensuring the participants’ complete willingness to participate, as well as the ability to complete the survey after seeing the doctor. The Arabic version of the DCF was piloted and Cronbach’s alpha was found to be 0.77.

### Sampling technique

Participants were allocated using a convenience sampling approach. Those patients in the waiting areas who fulfilled the aforementioned inclusion criteria were asked about their willingness to participate after the research team had provided a brief introduction about the study and confirmed their voluntary participation. Data were collected at any time (i.e., weekdays and weekends and morning, evening, and night shifts).

### Data management and analysis

The data were compiled using Microsoft Excel and then uploaded into the Statistical Package for the Social Sciences (IBM Corp. Released 2013, IBM SPSS Statistics for Windows, Version 22.0. Armonk, NY, USA) for analysis. Categorical variables (e.g., demographics) were represented as percentages and frequencies, while numerical variables (e.g., age) were summarized by calculating the mean and standard deviation. The prevalence, expressed in percentages, was calculated based on a 95% CI. Logistic regression was used to address the factors affecting patients’ visit to the ED. All results were considered statistically significant at *P* value < 0.05.

### Ethical considerations

This study was approved by the International Review Board of King Abdullah International Medical Research Center, Riyadh, Saudi Arabia. Participants were assured that their participation would be voluntary and anonymous and would not affect the health services provided to them. All DCFs were attached with a consent form that included the research purpose as well as the participants’ rights. All participants provided written informed consent to participate in this study.

## Results

### Baseline characteristics of study participants

The baseline characteristics of the respondents are shown in Table 1. Most participants were in the 20–39 age groups (62%). The mean age was 32.9 years with a standard deviation of 11.67 years. Male and female participants were equally represented in the study population. Regarding education, 12% of respondents did not complete high school and the rest either completed high school (40%) or had a bachelor’s degree or higher education (38%). Regarding occupation, 48% were unemployed, while 26% were employed in the military. Regarding marital status, 56% of the participants were married. Regarding income, 44% of respondents reported family income in the range 5000–9999 Saudi riyals, and 29% reported less than 5000 Saudi riyals. When asked about the chronicity of the presenting problem, only 30% said that the problem started on the day of the ED visit.

**Table 1:** Baseline characteristics of respondents

Variable	Category	*N*	%
Age group (years)	Mean ± SD (32.9 ± 11.67)		
	Under 20	27	9
	20–29	105	37
	30–39	71	25
	40–49	49	17
	50 and over	34	12
Sex	Male	165	50
	Female	163	50
Education	Did not complete high school	37	12
	Completed high school	128	40
	Diploma	33	10
	Bachelor and higher	120	38
Occupation	Unemployed	150	48
	Private work	13	4
	Health sector	12	4
	Military sector	80	26
	Government sector (not health)	31	10
	Private sector (not health)	17	6
	Other	7	2
Marital status	Married	180	56
	Single	121	37
	Widowed/divorced	23	7
Residence	In Riyadh	291	89
	Outside Riyadh	36	11
Family income/per month (Saudi Riyal)	< 5000	91	29
	5000–9999	139	44
	10,000–14,999	44	14
	≥ 15,000	39	13
When did this problem started?	Today	101	30
	Not today	233	70
Are you a patient waiting to be seen or a family member	Patient	147	46
	Family member	161	51
	Friend/co-worker	10	3
Do you have a primary care doctor or other health provider	No	232	84
	Yes	45	16
Did you try to call your primary care doctor before coming to the ED?	No	22	55
	Yes	18	45
If yes, what did the office say?	No appointments	4	24
	Too sick—need to go to ED	8	47
	Need further testing - that the doctor’s office cannot do	4	24
	Other	1	6
What is your main reason for coming to the emergency department?	Regular care here	85	29
	Excellence in care	127	43
	Insurance reasons	11	4
	Other financial reasons	13	4
	My doctor told me to come	30	10
	Close to where I live/work	29	10

*SD* standard deviation, *ED* emergency department

### The use of primary care by ED visitors

When asked whether they had a primary care provider (PCP), 84% answered “no.” Of the remaining 16% that answered “yes,” 45% tried to communicate with their PCP before visiting the ED. Of these, 47% were told they were too sick to be treated by the PCP and it was necessary to visit the ED.

### ED visitors’ expectations from the department

**Fig. 1:**
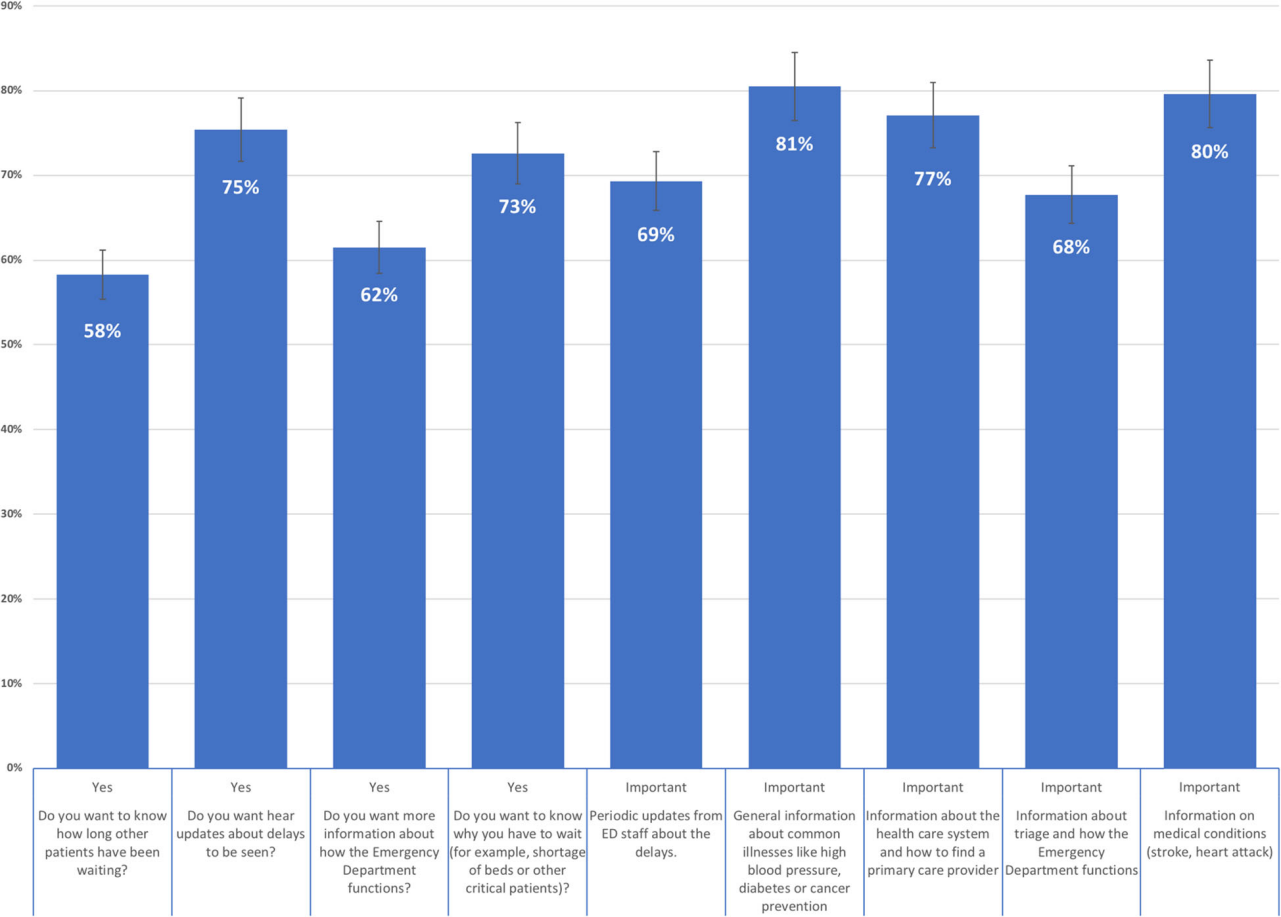
Patients expectations from the emergency department

Patients’ expectations from the ED are shown in Fig. 1. Of our respondents, 75% expressed their desire to be updated about any possible delays, and 73% wanted to know the cause of the delay. Additionally, 61% wanted more information on how the ED operates. Of these, 50% preferred the information to be delivered by a video playing in the waiting room, while 32% preferred handouts. The main reason for visiting the ED was excellence in care (43%) and having regular care in in the ED (29%). In terms of assessing the importance of various types of information provided during the waiting time, both general information about common illnesses (e.g., hypertension and diabetes) and information on serious medical conditions (e.g., stroke and heart attacks) were considered to be the most important by 81% and 80% of participants, respectively. Lastly, 77% of participants thought that it was important to know more information on the primary health care system and how to find a PCP.

### Knowledge of the participants about the triage system in the ED

Participants were asked if they knew why some patients are taken to the emergency room before others, even though they may not have waited as long. As shown in Table 2, 40% of respondents answered “no.” Among the 60% of participants who answered “yes,” 50% provided the correct explanation. Furthermore, most respondents (79%) viewed this action as fair, while only 21% viewed it as unfair. When asked about if they knew the definition of triage system, only 11% of participants answered in the affirmative and provided the correct answer; of the remaining participants, 76% answered “no,” or “yes” accompanied by an incorrect explanation. Lastly, participants were asked if they understood the function of a teaching hospital. Of 264 respondents, 71% answered “no” and 29% answered “yes.” Additionally, 46% were aware that they were attending a teaching hospital, while and the other 54% were not.

**Table 2:** Knowledge of the participant about the triage system in the emergency department

Variable	Category	*N*	%
Do you know why some patients are taken to a room before others even though they may not have waited as long?	Yes, with correct answer	170	51
	Yes, with incorrect answer or with no answer	32	10
	No	132	40
Do you think this is fair?	No	63	21
	Yes	236	79
Do you know what triage means?	Yes, with correct answer	37	11
	Yes, with incorrect answer or with no answer	43	13
	No	254	76
Do you know what a teaching hospital is?	No	187	71
	Yes	77	29
Do you know if this hospital is a teaching hospital?	No	72	54
	Yes	62	46

### The mean time in minutes patients expect to wait for results of medical investigations and processes undertaken in the ED

**Fig. 2:**
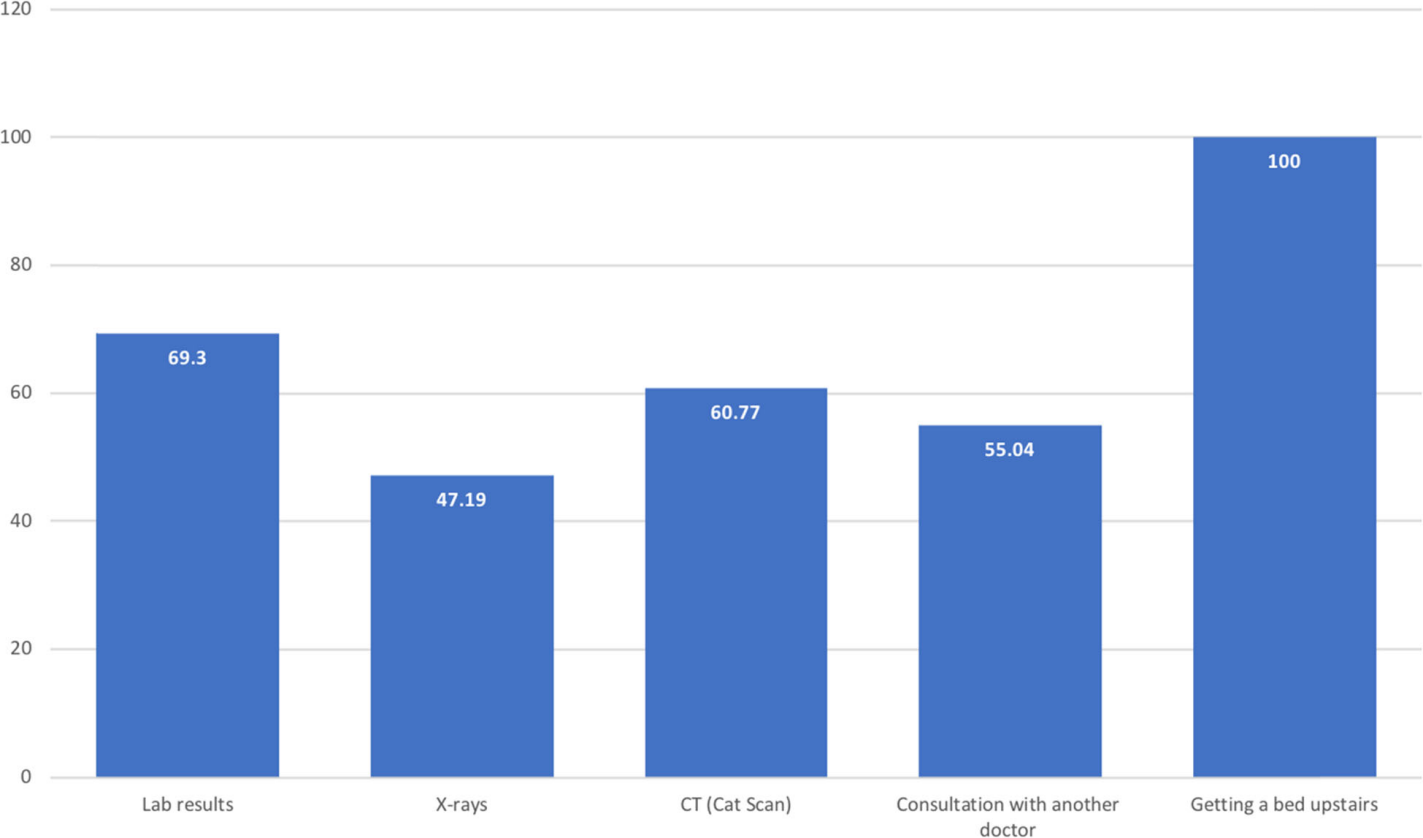
The mean time in minutes patients expect to wait for results of medical investigations and processes undertaken in the ED

As shown in Fig. 2, the mean expected time for receipt of laboratory results was 69.3 min. The mean time patients expected to wait for imaging findings varied depending on modalities (X-ray and CT), with patients expected X-ray results to take less time to deliver. Results of an X-ray were expected within a mean time of 47.19 min, while CT results were expected within a mean time of 66.77 min. Consultation with another doctor was expected to take a mean time of 55.04 min. Making arrangements to provide a bed upstairs in the patient’s home was expected to take the longest time, with a mean expectation of 100 min.

### Univariate logistic regression analysis of the factors affecting the triage knowledge

Participants who were under 20 years old were found to have a greater understanding of the triage system than those who were aged over 50 years (OR 2.21; 95% CI 0.44 to 11.11); however, this difference was not statically significant (*P* = 0.337). Moreover, there were no associations between patients’ sex, level of education, occupation, marital status, living place or family income, and the knowledge of the triage system. Table 3 summarized the results of the univariate logistic regression model for identification of the factors affecting knowledge about the triage system among ED visitors.

**Table 3:** Logistic regression analysis of factors affecting patients’ triage knowledge

Variable	Category	*P* value	OR	95% C. I for OR	
				Lower	Upper
Age group (years)	Under 20	0.337	2.21	0.44	11.11
	20–29	0.847	1.12	0.35	3.65
	30–39	0.368	1.65	0.55	4.94
	40–49	0.885	1.08	0.37	3.17
	50 and over*	1			
Sex	Male	0.077	0.51	0.24	1.08
	Female*	1			
Education	Illiterate	0.634	1.31	0.44	3.92
	Completed high school	0.857	1.07	0.52	2.17
	Diploma	0.522	1.36	0.54	3.43
	Bachelor and higher*	1			
Occupation	Unemployed	0.551	1.75	0.28	10.94
	Private work	0.178	4.55	0.50	41.34
	Health sector	0.947	0.92	0.09	9.39
	Military sector	0.374	2.45	0.34	17.60
	Government sector (not health)	0.726	1.44	0.19	11.25
	Private sector (not health)	0.078	6.87	0.81	58.52
	Other*	1			
Marital status	Married	0.514	0.71	0.25	2.00
	Single	0.884	0.91	0.26	3.23
	Widowed/divorced*	1			
Residence	In Riyadh	0.916	1.05	0.44	2.49
	Outside Riyadh*	1			
Family income (Saudi Riyal)	< 5000	0.423	1.50	0.56	4.06
	5000–9999	0.662	1.23	0.49	3.06
	From 10,000–14,999	0.93	0.96	0.34	2.69
	≥ 15,000	1			

*CI* confidence interval, *OR* odds ratio

*Reference group

## Discussion

Knowledge of triage systems among patients is not a widely investigated topic in the literature. The importance of addressing this issue lies in the fact that increased knowledge of triage systems is associated with increased patient satisfaction [10, 12, 13]. Consequently, it is important to gain an understanding of patient satisfaction as well as the level of ED visitors’ knowledge of the triage system and other factors that influence their experience during ED visits.

In this study, 61% of participants reported understanding why some patients are seen before others; of these participants, 83.6% where able to provide a correct explanation for their answers, while 16.4% failed to do so. These results are consistent with the findings of Seibert et al., who reported 68% of participants reported understanding this issue, of which 87% provided a correct explanation [14]. Those who understood why some patients are seen before others were more likely to consider this situation to be fair, an association that was also reported to be significant by Seibert et al. [14] After explaining triage to the participants, respondents in the study be Meek et al. reported a score of 6 on a 7-point scale for fairness [15]. Furthermore, 24% of participants reported an understanding of the triage process, with 45% of these participants were able to provide a correct explanation; thus, only 11% of participants were able to define triage correctly. This number is much lower than the 33% reported in the USA and the 50% reported in Australia [14, 15]. These results indicate that the population in Saudi Arabia is as likely as those of other counties to know why some patients are seen before others in the ED, yet less likely to be able to correctly define triage. Furthermore, our findings indicate that patient satisfaction in directly related to their level of knowledge about triage.

Although the services provided by EDs have undergone improvements, some variations have been noted. Such variations may prompt some patients to choose one ED and avoid another. For instance, distance from the ED and presumed waiting time were the most influential factors in dictating patients’ choice of ED [17]; however, in this study, excellence of care and having regular care in an ED were the main reasons that influenced patients’ decisions. Nearly 75% of patients stated the importance of receiving periodic updates about waiting time, with 69% of these preferring updates every 30 min. While 30 min was the shortest interval offered as an option in the present survey, the survey used by Cooke et al. included a 15-min interval option [18]. A greater proportion of respondents in that study still preferred updates every 30 min (55%) compared with every 15 min (21%). More than 80% of participants viewed the provision of information about health education topics (e.g., diabetes, stroke) during the waiting time as important, which is consistent with the findings of a study conducted in the Boston [19]. Lastly, 61% of participants wanted more information on how the ED operates, with 50% of these preferring the information to be delivered by a video playing in the waiting room. Educational videos during ED visits have been reported to be associated with increased patient satisfaction [20].

Primary health care (PHC) plays an integral role in the quality of every sector in the health care system, including EDs. Overall health in communities, reduction in mortality, and health inequalities can be improved by providing high-quality primary care services [21]. Accessing well-established PHC reduces ED’s visits by approximately 50% and referrals to specialty care by 30% [22]. Lack of regular access to PCPs is one of the reasons for ED overcrowding worldwide [23, 24]. An international study conducted by van den Berg et al. to investigate how the likelihood of attending an ED is related to accessibility of primary care showed the percentages of participants not having PCP in the following countries: 7.8% in Denmark, 10.7% in Belgium, 16.6% in Finland, 20.5% in England, 23.8% in Australia, 25.7% in Canada, 29% in Germany, 41% in Norway and Slovenia, 51.9% in Czech Republic, and 73.6% in Slovakia [25]. Those percentages, not surprisingly, were below that identified in this study in Saudi Arabia, with almost 84% of ED visitors reporting a lack of access to PHC. Moreover, only half of those who had PCPs in this study attempted to communicate with their doctor prior to attending the ED. This finding is not accordance with the study by Afilalo et al. showing that 22% of ED patients had tried to contact their PCPs before attending the ED [26]. Such apparent inconsistencies may be explained by variation in the health care systems in different countries. In Saudi Arabia, PHC was not a priority for the leading personalities in the health sector, which results in ED overcrowding. However, in 2016, the Saudi government announced a new plan (2030 vision) to encourage citizens to make use of PHC as a first step and by increasing family medicine residency training program opportunities all over the country [27].

The mean time patients expected to wait for test results reported by participants in this study did not vary significantly from that of other studies, although different approaches were used to assess this variable. In this study, after excluding outliers, the mean times patients expected to wait for the results of laboratory tests, X-rays, and CT scans were 69, 47, and 61 min, respectively, whereas Seibert et al. reported 60 min for laboratory test results, 35 min for X-rays, and 60 min for CT scans [15]. It is worth mentioning that the latter study reported the median expected times. In another study, 58% of patients reported expecting to receive their test results within 1 h [18].

In this study, we used a convenient sample of ED visitors at a one tertiary hospital in Riyadh. Therefore, our study population cannot be representative of all ED visitors in Saudi Arabia or the Middle East. Moreover, non-Arabic speakers were not asked to participate in this study, which further limits the generalizability of our findings. Furthermore, variations in the work pressure and patient flow in the ED with time might influence our findings. However, the data were collected during different shifts (morning, evening, and night) on weekdays and weekends. Furthermore, the results could be skewed by the response rate of 80% and differences in patient waiting times during which the DCF was filled.

## Conclusion

In conclusion, our population is not aware of triage. Efforts should be made to promote knowledge of triage. Additionally, only a small percentage of respondents said they have a primary care provider. It is important to provide access to primary care to community members. Finally, respondents in this study wanted more information regarding their visit and general information about health. These expectations should be met either by public health campaigns or educating material within the ED.

## Data Availability

Please contact author for data requests.

## Abbreviations

CI: Confidence interval
CT: Computerized tomography
DCF: Data collection form
ED: Emergency department
IQR: Interquartile range
KAMC-ED: King Abdulaziz Medical City – Emergency Department
OR: Odds ratio
PCP: Primary care provider
PHC: Primary health care
SD: Standard deviation

## References

[1] Rehmani R, Norain A (2007). Trends in emergency department utilization in a hospital in the Eastern region of Saudi Arabia. Saudi Med J.

[2] Moore BJ, Stocks C, Owens PL (2017). Trends in emergency department visits, 2006–2014.

[3] Unwin M, Kinsman L, Rigby S (2016). Why are we waiting? Patients’ perspectives for accessing emergency department services with non-urgent complaints. Int Emerg Nurs.

[4] Dawoud SO, Ahmad AMK, Alsharqi OZ, Al-Raddadi RM (2015). Utilization of the emergency department and predicting factors associated with its use at the Saudi Ministry of Health General Hospitals. Glob J Health Sci.

[5] Alyasin A, Douglas C (2014). Reasons for non-urgent presentations to the emergency department in Saudi Arabia. Int Emerg Nurs.

[6] Villanueva CA, Almadani M, Mahnashi F, Alyhya S, Alshreef O (2017). Waiting time in emergency department in Riyadh 2017. J Biosci Med.

[7] Moskop JC, Sklar DP, Geiderman JM, Schears RM, Bookman KJ (2009). Emergency department crowding, part 1—concept, causes, and moral consequences. Ann Emerg Med.

[8] Qureshi NA (2010). Triage systems: a review of the literature with reference to Saudi Arabia. East Mediterr Health J.

[9] Bruijns SR, Wallis LA, Burch VC (2008). Effect of introduction of nurse triage on waiting times in a South African emergency department. Emerg Med J.

[10] Miles Jeffrey A., Naumann Stefanie E. (2004). The English Patient: A Model of Patient Perceptions of Triage in an Urgent Care Department in England. M@n@gement.

[11] Bleustein C, Rothschild DB, Valen A, Valatis E, Schweitzer L, Jones R (2014). Wait times, patient satisfaction scores, and the perception of care. Am J Manag Care.

[12] Shah S, Patel A, Rumoro D, Hohmann S, Fullam F (2015). Managing patient expectations at emergency department triage. Patient Exp J.

[13] Thompson DA, Yarnold PR, Williams DR, Adams SL (1996). Effects of actual waiting time, perceived waiting time, information delivery, and expressive quality on patient satisfaction in the emergency department. Ann Emerg Med.

[14] Seibert T, Veazey K, Leccese P, Druck J (2014). What do patients want? Survey of patient desires for education in an Urban University Hospital. West J Emerg Med.

[15] Adeniji AA, Mash B (2016). Patients’ perceptions of the triage system in a primary healthcare facility, Cape Town, South Africa. African J Prim Heal Care Fam Med.

[16] Meek R, Phiri W (2005). Australasian triage scale: consumer perspective. Emerg Med Australas.

[17] Grafstein E, Wilson D, Stenstrom R, Jones C, Tolson M, Poureslami I (2013). A regional survey to determine factors influencing patient choices in selecting a particular emergency department for care. Acad Emerg Med.

[18] Cooke T, Watt D, Wertzler W, Quan H (2006). Patient expectations of emergency department care: phase II--a cross-sectional survey. CJEM.

[19] Kit Delgado M, Ginde AA, Pallin DJ, Camargo CA (2010). Multicenter study of preferences for health education in the emergency department population. Acad Emerg Med.

[20] Papa L, Seaberg DC, Rees E, Ferguson K, Stair R, Goldfeder B (2008). Does a waiting room video about what to expect during an emergency department visit improve patient satisfaction?. CJEM.

[21] Starfield B, Shi L, Macinko J (2005). Contribution of primary care to health systems and health. Milbank Q.

[22] Now More Than Ever Universal coverage reforms service delivery reforms, leadership reforms, public policy reforms. The World Health Report. Primary Health Care. The World Health Organization; Accessed 11 Apr 2019; Available at: https://www.who.int/whr/2008/whr08_en.pdf.

[23] Wexler R, Hefner JL, Sieck C, Taylor CA, Lehman J, Panchal AR (2015). Connecting emergency department patients to primary care. J Am Board Fam Med.

[24] Redstone P, Vancura JL, Barry D, Kutner JS (2008). Nonurgent use of the emergency department. J Ambul Care Manage.

[25] van den Berg MJ, van Loenen T, Westert GP (2016). Accessible and continuous primary care may help reduce rates of emergency department use. An international survey in 34 countries. Fam Pract.

[26] Afilalo J, Marinovich A, Afilalo M, Colacone A, Léger R, Unger B (2004). Nonurgent emergency department patient characteristics and barriers to primary care. Acad Emerg Med.

[27] Caring for our health | Saudi Vision 2030. Accessed 11 Apr 2019; Available at: https://vision2030.gov.sa/en/node/68

